# Highly loaded deoxypodophyllotoxin nano-formulation delivered by methoxy polyethylene glycol-block-poly (D,L-lactide) micelles for efficient cancer therapy

**DOI:** 10.1080/10717544.2020.1716875

**Published:** 2020-01-31

**Authors:** Chang Zu, Yinglan Yu, Caiwei Yu, Yi Li, Runing Sun, Birendra Chaurasiya, Baoqiang Tang, Daquan Chen, Jiasheng Tu, Yan Shen

**Affiliations:** a Department of Pharmaceutics, China Pharmaceutical University, Nanjing, China;; b School of Pharmacy, Yantai University, Yantai, China;; c School of Engineering, China Pharmaceutical University, Nanjing, China

**Keywords:** Deoxypodophyllotoxin, mPEG-PDLLA, polymeric micelles, lyophilization, anti-tumor

## Abstract

Cancer is a kind of malignant diseases that threatens human health and the research application of anti-tumor drug therapeutics is growingly always been focused on. Many new compounds with great anticancer activity were synthesized but cannot be hard to be developed into clinical use due to its poor water solubility. Deoxypodophyllotoxin (DPT) is just an example. We develop lyophilized Deoxypodophyllotoxin (DPT) loaded polymeric micelles using methoxy polyethylene glycol-block-Poly (D, L-lactide) (mPEG-PLA). DPT-PM freeze-dried powder was successfully prepared using optimized formulation. mPEG-PLA was added to hydration media before hydrating as cryoprotectants. The freeze-dried powder exhibited white pie-solid without collapsing, and the particle size of DPT-PM reconstituted with water was about 20-35 nm. The entrapment efficiency of the reconstituted solution was 98%, which shows no differences with the micelles before lyophilization. *In-vitro* cytotoxicity and cellular uptake studies showed that DPT-PM has a higher degree of cytotoxicity comparing with DPT and mPEG-PLA micelles and uptake of mPEG-PLA was concentration and time-dependent. In vivo characterization of DPT-PM was done for pharmacokinetics behaviors, antitumor activity and safety. The obtained results showed significant improvement in plasma clearance bioavailability (*p* <0.05) and prolonged blood circulation time comparing with DPT-HP-β-CD. Moreover, mPEG-PLA micelles had a better degree of anti-tumor efficacy, this was due to better accumulation of mPEG-PLA in tumor cell via enhanced permeability and retention (EPR) effect. Therefore, DPT-PM has great clinical value, and can be expected to be a novel antitumor preparation.

## 1. Introduction

**Figure 1.**
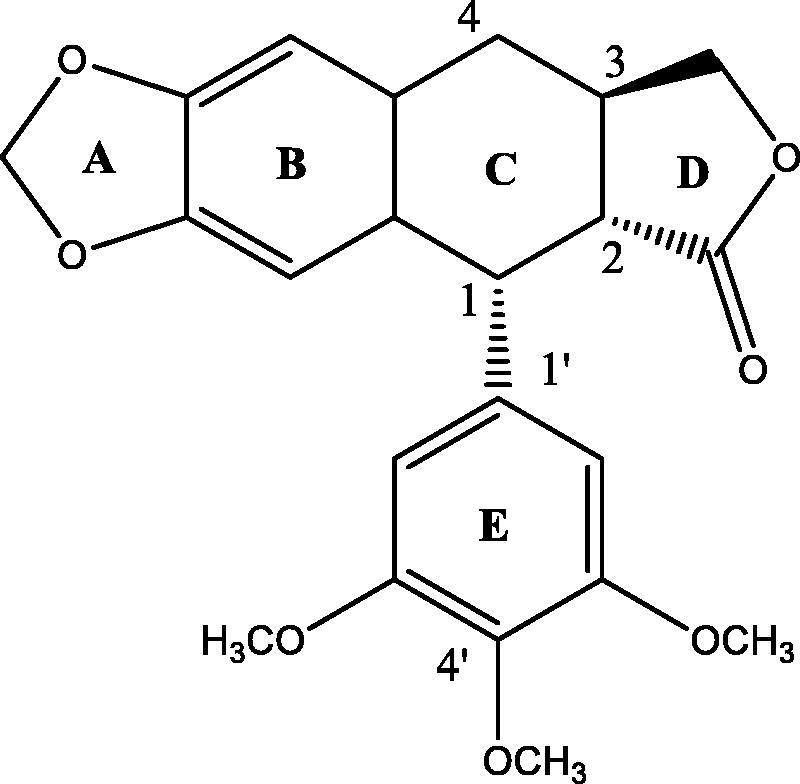
Chemical structure of deoxypodophyllotoxin.

Podophyllotoxin, was first isolated in 1880 by Podwyssotzki from the North American plant Podophyllum peltatum L. (American podophyllum) (Canel et al., 2000). Podophyllotoxin binds with tubulin and arrest the microtubules assembly and thus, stop cell cycle in metaphase (Bohlin & Rosen, 1996; Canel et al., 2000; Yong et al., 2009). Unfortunately, podophyllotoxin has failed in clinical practice. Moreover, it can be extremely dangerous in case of overdose. Deoxypodophyllotoxin (DPT, Figure 1), is considered as Anthriscus sylvestris L. Hoffm’s main lignan constituent (Gordaliza et al., 2000). It has several pharmacological properties including antiproliferative, antitumor, antiviral, anti-inflammatory, anti-platelet aggregation and anti-allergic (Dall’Acqua et al., 2006; Chen et al., 2011; Zilla et al., 2014; Hui et al., 2016). It has potent anti-proliferative and anti-tumor effects against broad varieties of cancers by modulating the microtubule (Chaudhuri & Luduena, 1996; Guerram et al., 2015). As a promising microtubule inhibitor, the clinical applications of this molecule were limited by its hydrophobicity.

In order to overcome this problem, many scientists have done related research. The representative methods include liposome (Chen et al., 2006; Ikeda et al., 2011), micronization (Feng et al., 2014; Karwal et al., 2016), solubilizers (Vanuden et al., 1994), cyclodextrin inclusions, self-emulsifying drug delivery systems polymer micelles (Fan et al., 2009; Wang et al., 2016) and so on. DPT and its intravenous formulation of β-cyclodextrin inclusion complex have been adopted for phase I evaluation. As a poorly soluble drug carrier, polymer micelles have the following advantages: (1) increase the drug solubility and decrease the therapeutic dose; (2) wrap the drug inside the carrier to avoid degradation and inactivation of the drug and reduce toxic and side effects; (3) Because the micelle size is below 100 nm, it can avoid phagocytosis of the reticuloendothelial system (RES) and prolong the circulation time; (4) Good biodegradability and biocompatibility; (5) High permeability and retention (enhanced permeation and retention, EPR effect), to achieve passive targeting; (6) Polymer molecular weight can also avoid kidney clearance and sterilization operations (Burt et al., 1999; Gaucher et al., 2005).

Polyethylene glycol monomethyl ether-polylactic acid block copolymer (Methoxy Polyethylene Glycol-block-Poly (D, L-lactide, mPEG-PLA) is one of the most commonly used amphiphilic copolymers (Yu et al., 2018). Its hydrophilic segment is PEG, which is the most widely used shell material in polymer micelle delivery systems due to its highly hydrated, an efficient steric protector, and good biocompatible, and it has low toxicity. The core-forming molecules are PLA, and the final metabolite in the body is carbon dioxide and water, which has been approved by the US FDA as a hydrophobic block for drug controlled release dosage carriers (Oerlemans et al., 2010). In an aqueous environment, both hydrophilic end (mPEG) and the hydrophobic end (PLA) exposed out in a large number and form an aqueous protective layer outside of the micelles. The hydrophobic moiety PLA binds with hydrophobic drug and encapsulates in the core of the micelle and increases its solubility. The safety evaluation of this polymer was conducted by HM Burt e.al (Bissery, 1995). and reported that it has no systemic as well as local toxicities. Due to its good safety profile and high drug loading affinity, this polymer is widely used as pharmaceutical excipients in the preparation of verities of dosage-form especially in the parenteral formulation. This is also approved by the FDA to be used as an excipient in pharmaceutical preparation (Yang and Kao, 2006).

In spite of many attractive advantages of polymeric micelles as promising drug delivery systems, one drawback is their physical instability. As micelles are in the nanosize range and more dynamic than polymeric nanoparticles, they exhibit greater aggregation tendency due to kinetic motion. Upon storage and transportation, drug leakage may take place due to diffusion of the drug outward the micelles during fluctuation of temperature. One approach to overcome such problems is the complete elimination of water by lyophilization into a dried powder form. Consequently, their shelf-life can be extended by preventing aggregation of the system and leakage of the encapsulated drug. Therefore, this study aimed to develop lyophilized deoxypodophyllotoxin-polymeric micelles (DPT-PM) (Yu et al., 2018). The physicochemical characteristics of the polymeric micelles were investigated in terms of particle size, entrapment efficiency, and drug loading. In addition, in order to characterize the physicochemical properties of micelles and lyophilized powders, the in vitro release behavior of the micelles were investigated. The in vitro cytotoxicity assay and *in vitro* cell uptake assay were used to evaluate the efficacy of the assay. The fluorescence *in vivo* imaging experiments were used to investigate the distribution of micelles in mice.

## 2. Methods

mPEG (Mn = 2000; Fluka, MO) and PLA were purchased from Jinan Daigang Biomaterial Co. Ltd. (Shang Dong, China). mPEG-PLA (50:50) was self-synthesized (Yu et al., 2018). DPT and DPT-HP-β-CD were obtained from the Institute of Pharmaceutical Chemicals, China Pharmaceutical University, China.

Hela229 cells purchased from Nanjing Keygen Biotech Co., Ltd.

Female SD and nude mice were obtained from Qinglongshan farms (Nanjing, China). All animal experiments complied with the requirements of the National Institute of Health Guide for Care and the procedures were approved by the China Pharmaceutical University Animal Experiment Center.

### 2.1. Preparation of DPT-PM

**Scheme 1.**
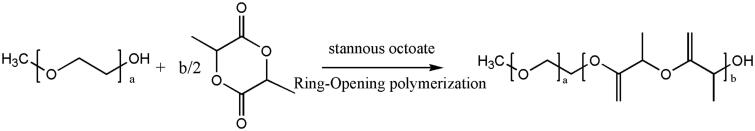
mPEG-PLA reaction equation.

DPT-PM was prepared by the solvent evaporation-film dispersion method. Briefly, the desired amounts of DPT (0.6 g) and mPEG-PLA (3.0 g) were dissolved in a certain amount of methanol in round-bottomed flask. The alcoholic content of the preparation was removed in a rotary evaporator at 40 °C under reduced pressure. The formed film inside the flask was kept for 5 min in untouched condition than after 60 mL of water was poured into to suspend the film and was passed through the membrane filter (0.22 mm) to get DPT-PM. The reaction equation is shown in Scheme 1.

### 2.2. Optimization of cryoprotectant

The optimization method of cryoprotectant that prepared lyophilization powder of DPT-PM without cryoprotectant, and then added sucrose, lactose, trehalose, sorbitol, mannitol, glycine, PEG2000 Poloxamer 188, mPEG-PLA by the addition method as cryoprotectants. The concentration of 5% DPT-PM. Comparing the appearance of lyophilized powder, its reconstitution, particle size and entrapment efficiency (%), a suitable lyophilized protective agent was screened. The optimal concentration of cryoprotectant was selected by adding different concentrations of cryoprotectant (2, 3, 4, 5, 6, 8, 10%) in the preparation of DPT-PM lyophilized powder. It was screened by comparing the appearance and reconstitution of lyophilized powder.

To measure the encapsulation and loading efficiency of DPT-PM, it was dissolved in water and diluted with methanol at 0.1 mg/mL concentration. Analyses were performed by HPLC method using Hedera C18 column (4.6 mm × 250 mm, 5 µm). The column oven temperature was maintained at 30 °C. The methanol–water (75:25 *v/v*) was used as the mobile phase and flow rate was maintained at 1.0 mL/min, the sample injection volume was fixed at 20 µL. The signal was monitored at 294 nm with UV detector. The encapsulation efficiency (*EE*) and drug loading efficiency (*DL*) of the DPT-PM were calculated by using the following equation:

$$EE(\%)=\frac{W_{A}}{W_{B}}\times 100\%$$

$$DL(\%)=\frac{W_{A}}{W_{C}}\times 100\%$$

*W_A_* represents the amount of DPT-loaded in the DPT-PM, *W_B_* represents the total DPT amount added during preparation of the DPT-PM and *W_C_* represents the weight of the DPT-PM.

Screening of cryoprotectants addition methods, using internal and external addition of the best cryoprotectant to prepare DPT-PM lyophilized powder. It was screened by comparing the particle size, reconstitution of lyophilized powder and encapsulation efficiency.

### 2.3. Characterization of DPT-PM

#### 2.3.1. Morphology

The morphology of DPT-PM solution was observed by transmission electron microscopy (TEM, JEOL, JEM-1400; Japan). The sample was prepared by placing a drop of DPT-PM (dilute 50-fold with double-distilled water) on a 400-mesh copper grid coated with carbon film followed by negative staining with 1.5% (*w/v*) phosphotungstic acid. Then, the sample was dried in the air before TEM observation.

#### 2.3.2. Particle size analysis

Particles size and size distribution of DPT-PM were measured by dynamic light scattering (DLS) using a laser particle size analyzer (ZetaPALS, Brookhaven Instruments Co., USA). Before measuring the particle size, the sample was dispersed into saline. The measurement was repeated for three times at 25 °C.

#### 2.3.3. DSC analysis

Differential scanning calorimetry (DSC) analysis was performed on a DSC204 (NETZSCH, Germany). 15 mg sample was placed in an aluminum pan and was sealed with the sample pan press. The probes were heated from 30 to 200 °C at a rate of 10 °C/min under nitrogen atmosphere.

#### 2.3.4. In vitro release profile

The *in vitro* release profile of DPT-PM was conducted in phosphate buffer saline at pH 7.4 containing 1% polysorbate 80. A certain amount of DPT-PM (DPT about 1 mg) was directly placed into dialysis bags (5.0 cm × 2.5 cm, MWCO =3500 kDa) and was sunk in 100 mL of release medium and was placed into horizontal oscillating water bath at 37 °C and oscillated at 75 rpm. 1 mL sample was drawn and replaced with the release medium at a time interval of 1, 2, 4, 6, 8, 12, 24, 36 and 48 h. Collected samples were analyzed by HPLC method to determine the concentration of DPT.

Investigate the release of DPT-PM at different pH conditions in vivo. Select pH 7.4, 6.8 and 5.0 PBS buffers as release media to simulate the blood environment (pH 7.4) and tumor extracellular (pH 6.5–7.0), as well as endosomic acid environment (pH 5.0), 1% PBS was added to meet the sink condition.

#### 2.3.5. Cytotoxicity study

Human cervical cancer cell line (Hela) was purchased from the Chines Academy of Science (Shanghai, China). The cells were cultured in DMEM cell culture medium containing 10% fetal bovine serum (FBS), 50 U/mL penicillin, 50 μg/mL streptomycin and 1% l-glutamine in a humidified incubator at 37 °C with 5% CO_2_.

The cells were seeded at a density of 10 × 10^3^ cells/well in a 96-well plate and incubated for 24 h. DPT, mPEG-PLA and DPT-PM were serially diluted with culture medium in a concentration of 1 × 10^−6^, 1 × 10^−7^, 1 × 10^−8^, 1 × 10^−9^ and 1 × 10^−10  ^mol/L. After 24 h of cell growth, cell culture medium from each well was replaced with 200 μL of diluted samples and the cells were incubated for 24 h cytotoxicity study. Then after, samples were gently aspirated and 20 μL of MTT solution (5 mg/mL) was added into each well and incubated into an incubator for 4 h. After incubation for 4 h, MTT solution was replaced by 150 μL of dimethyl sulfoxide (DMSO) and was gently sacked for 5 min to dissolve formazan. The absorbance of formazan was recorded at 570 nm in Microplate Reader. All of the experiments were performed in triplicates. Cell inhibition percentage was calculated as following:

$$\text{Cell}\;\text{inhibition}\;\text{rate}=1-\frac{I_{\mathrm{sample}}-I_{\mathrm{blank}}}{I_{\mathrm{control}}-I_{\mathrm{blank}}}\times 100\%$$

*I_sample_*: the absorbance of formazan in live cells incubated with drug; *I_blank_*: the absorbance of comparison; *I_control_* is the mean absorbance value of the control group.

#### 2.3.6. The cell uptake study

Hela cells were seeded in a 6 well plate at a density of 5 × 10^5^ cells/well and allowed to attach for 24 h. After the proper adherence of cells on the bottom of the well, the culture medium was aspirated and washed three times with PBS buffer. 0.1, 0.5, 2, 5 and 10 μL of mPEG-PLA micelle solution coated with100 ng/mL coumarin 6 was added respectively in a set of three wells and incubated for 1 h in incubator. Then after, the medium was aspirated and washed three times with cold PBS (4 °C). 0.5 mL of trypsin was added into each well to harvest the adhered cells and was centrifuged to separate the cells. Further, the settled cells were suspended into 0.5 mL cold PBS and the up-take of coumarin 6 tagged samples were measure by flow cytometry (Ex = 488 nm; Em = 530 nm) (MACSQuant Analyzer 10, Miltenyi Biotec GmbH).

#### 2.3.7. Tissue distribution

For tissue distribution study, Hela229 tumor cell line (5 × 10^5^ cell/200 μL) was injected into the right-limb flank of the nude mice (weight : 20±2 g, *n* = 12). After 2 weeks of tumor growth, all mice were divided into two groups (*n* = 6). One group was injected with 200 μL of DiR-labeled DPT-PM via tail vein and another group was injected with free DiR at a fixed DiR dose of 1.25 mg/kg in both groups. After injection, both groups were imaged under *in vivo* imaging system (*In-Vivo* FX PRO, Carestream, Canada) at the time interval of 1, 2, 4, 6 and 8 h. After 8 h of imaging, one mouse from each group was sacrificed to harvest the tumor and major organs (heart, liver, spleen, lung and kidney) and was imaged under IVIS and the obtained fluorescence intensities were calculated.

#### 2.3.8. Pharmacokinetic study

SD female mice (weight : 230 ± 10 g) were used for *in vivo* experiments. All mice were grouped into two groups (*n* = 12). One group was injected with 0.55 mL of DPT-PM solution and another group was injected with hydroxypropyl -β- cyclodextrin solution. The dose given to each mouse was calculated based on the bodyweight of the mouse (15 mg/kg DPT). The blood samples were collected from eye by puncturing the optical vain plexus at a predetermined time interval of 2, 5, 10, 20, 30, 45, 60, 90, 120, 180 and 240 min.

To determine the concentration of DPT, 100 μL of plasma sample was taken and was mixed with 10 μL of diazepam (internal standard) for 10 s and 300 μL of methanol was added into it and was further vortex for 3 min. The mixed samples were centrifuge at 14000 rpm for 5 min and 20 μL from the supernatant was injected into the HPLC system to quantify the amount of DPT.

#### 2.3.9. Statistical analysis

All experiments were conducted in triplicate and the data were analyzed using SPSS software, version 19. Differences between the groups were assessed by one-way ANOVA, and a *p* value less than 0.05 was considered as significant.

## 3. Results

### 3.1. Optimization of cryoprotectant

The factors influencing the cryoprotectant of DTX-PM were optimized by using a single-factor design. Three variable factors were chosen for the analysis: (A) different cryoprotectants; (B) addition method of cryoprotectants; (C) concentration of cryoprotectants.

As shown in Table 1, different cryoprotectants were optimized for their dispersion efficiency, particle size, and entrapment efficiency. Without cryoprotectant, PEG 2000 appeared as a white collapsed mass which upon addition of water precipitated and was difficult to disperse. The entrapment efficiency of Glycine, Poloxamer188 and mPEG-PLA was more than 90%, which shows nearly no differences with the micelles before lyophilization (*p* >0.05). On the other hand, the addition of mPEG-PLA did not significantly affect the particle size of DPT-PM as compared to those without a stabilizer (*p*>0.05). From these results, mPEG-PLA was chosen as a stabilizer for the lyophilization, since it did not change the particle size and %EE of DPT-PM.

**Table 1.** Effects of different cryoprotectant on DPT-PM freeze-dried powder (*n* = 3, mean ± SD).

Cryoprotectant	Redispersion Time (s)	Particle size (nm)		Entrapment efficiency (%)		Appearance of dry powers
		Before	After	Before	After	
None	—	28.6	—	99.46	8.76	White mass collapsed
Sucrose	150	29.4	126.7	98.89	32.93	White mass non-collapsed
Lactose	150	30.2	158.5	99.21	13.21	White mass non-collapsed
Trehalose	100	26.5	83.6	99.32	88.42	White mass non-collapsed
Sorbitol	60	38.1	52.8	98.67	88.31	White mass non-collapsed
Mannitol	180	27.9	182.4	99.51	15.58	White mass non-collapsed
Glycine	45	30.4	38.6	99.63	90.25	White mass non-collapsed
PEG2000	—	34.6	—	99.42	10.21	Nearly white collapsed
Poloxamer188	90	27.3	54.3	99.13	92.31	White mass non-collapsed
mPEG-PLA	30	28.9	32.2	99.41	97.28	White mass non-collapsed

Similarly, the addition method of cryoprotectant was optimized. The obtained result shows there was no significant difference in terms of particle size and %EE (Table 2). While comparing particle size in both methods, it was seen that there was a slight difference in the obtained particles size. It was seen that after the addition of cryoprotectant in hydration condition particles were obtained with smaller size than was added before freeze-drying. Based on this, it was concluded that the best way to add cryoprotectant is in hydrate condition.

**Table 2.** Effects of added method of cryoprotectant on DPT-PM freeze-dried powder.

Added method	Redispersion time (s)	Particle size (nm)		Entrapment efficiency (%)	
		Before	After	Before	After
Added before freeze drying	30	28.9	32.2	99.41	97.28
Added to hydration media	5	30.1	29.2	99.67	99.13

Likewise, the effect of concentration of cryoprotectant on the size of particles and the %EE was studied. The obtained results show (Table 3) that the optimum concentration of the cryoprotectant required for freeze-drying was 3% at which no significant changes in particles size and %EE was recorded.

**Table 3.** Effects of concentration of cryoprotectant on DPT-PM freeze-dried powder.

Concentration of mPEG-PLA as cryoprotectant (%)	Redispersion time (s)	Particle size (nm)		Entrapment efficiency (%)	
		Before	After	Before	After
2	10	28.2	45.2	99.32	96.29
3	5	32.4	31.9	99.72	98.96
4	5	27.6	28.0	98.76	98.72
5	5	30.1	29.2	99.67	99.13
6	5	24.2	25.1	99.35	99.32
8	5	25.6	22.4	99.48	99.21
10	5	31.8	27.3	98.72	97.53

In summary, DPT-PM freeze-dried powder was successfully prepared using optimized formulation. mPEG-PLA was added to hydration media before hydrating as cryoprotectants. The freeze-dried powder exhibited white pie-solid without collapsing, and the particle size of DPT-PM reconstituted with water was about 20–35 nm. The entrapment efficiency of the reconstituted solution was 98%, which shows no difference with the micelles before lyophilization. DPT-PM was obtained with a saturation solubility of 18.18 mg/mL, which was 36,000 times higher than that of the raw DPT.

### 3.2. Characterization of DPT-PM

**Figure 2.**
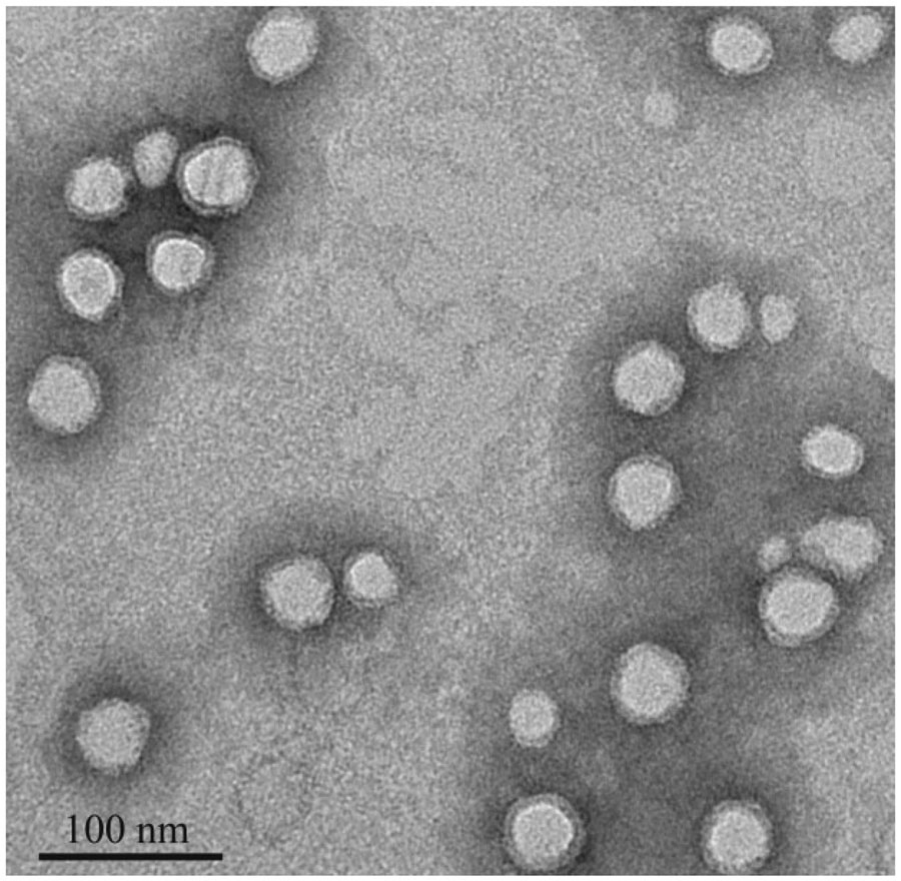
TEM images of DPT-PM.

The morphology of micelles was characterized by TEM (Figure 2). All micelles exhibited almost spherical in shape, uniformly distributed and smaller in size (13–30 nm). From the TEM image, it was clearly seen that all micelles were enveloped in a coronal layer which provides stability to micelles (Xiao et al., 2018).

**Figure 3.**
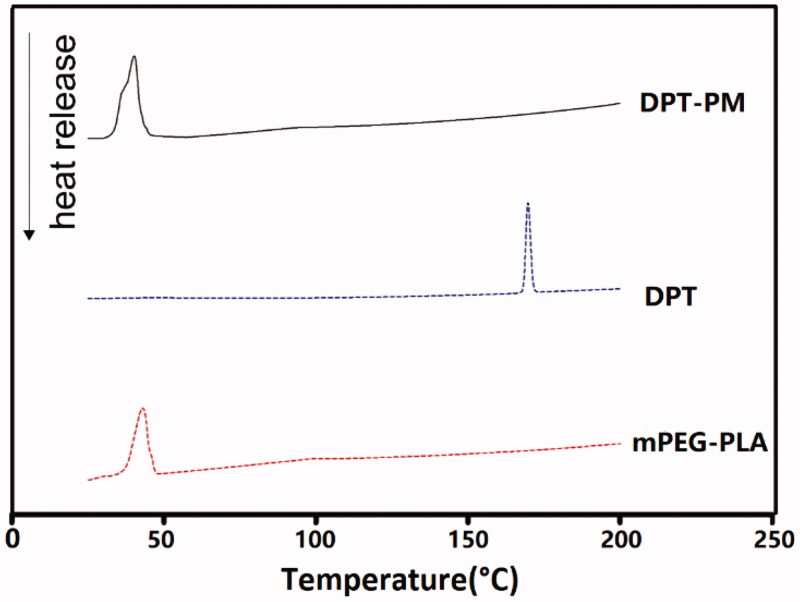
DSC thermograms of DPT-PM、DPT and mPEG-PLA.

DPT raw material (Figure 3) was monitored at 169.69 °C and in micelle at 40.14 °C. After freeze-drying with mPEG-PLA, the peak was monitored at 42.1 °C with a slight shift. The nature of peak was not changed in micelle as well as in freeze-dried powder. From this study, it was conformed that there was no incompatibility of DPT with polymer and cryoprotactent and its crystalline property was intact even after lyophilization. Drug loading and entrapment efficiency of PM is higher in compression to HP-β-CD. The calculated loading and entrapment % of DPT in solution and in freeze-dried powder with PM and in HP-β-CD were listed in Table 4.

**Table 4.** Drug loading and entrapment efficiency of DPT-PM solution and powder (*n* = 3, mean ± SD).

	DPT-PM solution	DPT-PM freeze-dried powder	DPT-HP-β-CD solution
Drug loading%	19.5 ± 0.02	19.74 ± 0.10	3.40 ± 0.01
Entrapment efficiency%	99.3 ± 0.53	98.75 ± 0.66	80.2 ± 0.25

#### 3.2.1. In vitro release studies

**Figure 4.**
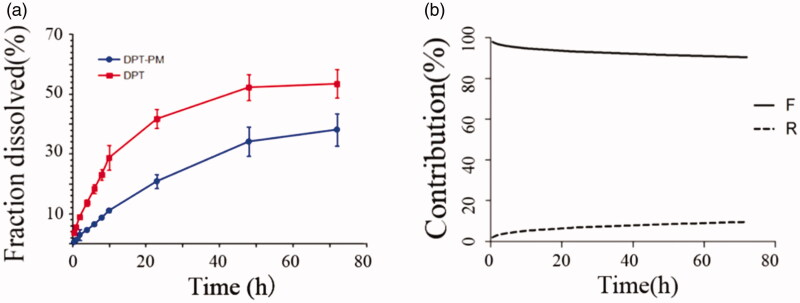
Cumulative release profile of DPT-PM and DPT at pH 7.4 (a); percentage of the Fick diffusion and the erosion mechanisms over the drug release from DPT-PM (b).

The release profiles of DPT from micelle DPT-PM and plane DPT at pH 7.4 were shown in Figure 4(a). From release profile, it can be seen that the cumulative release of DPT was higher (53.4%) from DPT-PM and lower (38.1%) as such after 72 h. From this in-vitro drug release profile study, it can be predicted that the release of DPT in plasma environment will be higher from micelles (DPT-PM) than from DPT as such. From release pattern, it can also be predicted that the release of DPT from PM is in controlled form and last for a long time. The release of DPT was fitted into different release models using DDsolver software and the calculated results were listed in Table 5.

**Table 5.** Fitting equation and correlation coefficients.

Model	Equation	*R*^2^
Zero-order	Q = 0.726 t	0.4219
First-order	Q = 100[1−Exp(−0.015t)]	0.7736
Higuchi	Q = 6.724t^0.5^	0.9458
Peppas-Sahlin	Q = 6.493t^0.664^–0.165t^1.328^	0.9957

After fitting the release pattern of DPT into different models, it was clear that the release pattern follows the Peppas–Sahlin model as the *R*^2^ value obtained was 0.9957.

From this Peppas–Sahlin model, it can be said that the release mechanism coexists diffusion and dissolution but not the Fick-diffusion mechanism.

It can be seen from Figure 4(b), the drug release pattern was dominated by diffusion mechanism throughout the release process. But it can be also seen that a portion of the drug gets dissolved slightly in the initial phase and further follow the diffusion mechanism. From the graph, it can be seen that only 9.51% of the drug released from the matrix by dissolving in release media in 72 hours. From the plotted graph, it can be concluded that the dissolution mechanism increases by the passes of time, however, in comparison to diffusion mechanism it is always less in proportion. Based on this speculation, it can be said that DPT slowly released from the shell formed outside by mPEG-PLA by diffusion mechanism initially and later by penetration of release medium into shell followed by dissolving the core materials. From this, it is clear that the release of DPT from mPEG-PLA shell was driven by two mechanisms which acts synergistically.

**Figure 5.**
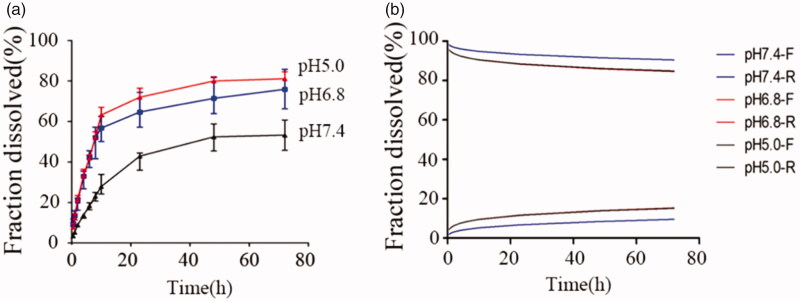
Cumulative release profile of DPT from DPT-PM at pH 5.0, 6.8 and 7.4 (a); percentage of the Fick diffusion and the erosion mechanisms over the drug release from DPT-PM at different pH (b).

Further, to confirm the influence of release medium pH of release pattern, the release of DPT from micelles was conducted at 3 different pH levels i.e. pH 7.4, 6.8 and 5.0. The obtained results at different pH levels were calculated and fitted into Peppas–Sahlin model and the calculated values were listed in Table 6 and the release profile was shown in Figure 5. From the cumulative drug release profile, it was clear that the release of DPT from mPEG-PLA is higher at acidic pH. As the tumoral microenvironment is acidic, so it is concluded that DPT gets released in tumor cell in higher concentration and not in normal cell.

**Table 6.** Peppas-Sahlin equation at different pH.

pH	Equation	*k*_1_	*k*_2_	*m*	*R*^2^
7.4	Q = 6.493 t^0.664^−0.165t^1.328^	6.493	−0.165	0.664	0.9957
6.8	Q = 19.160 t^0.556^−1.060t^1.112^	19.160	−1.060	0.556	0.9882
5.0	Q = 17.912t^0.542^−1.002t^1.084^	17.912	−1.002	0.542	0.9779

### 3.3. The cytotoxicity study

**Figure 6.**
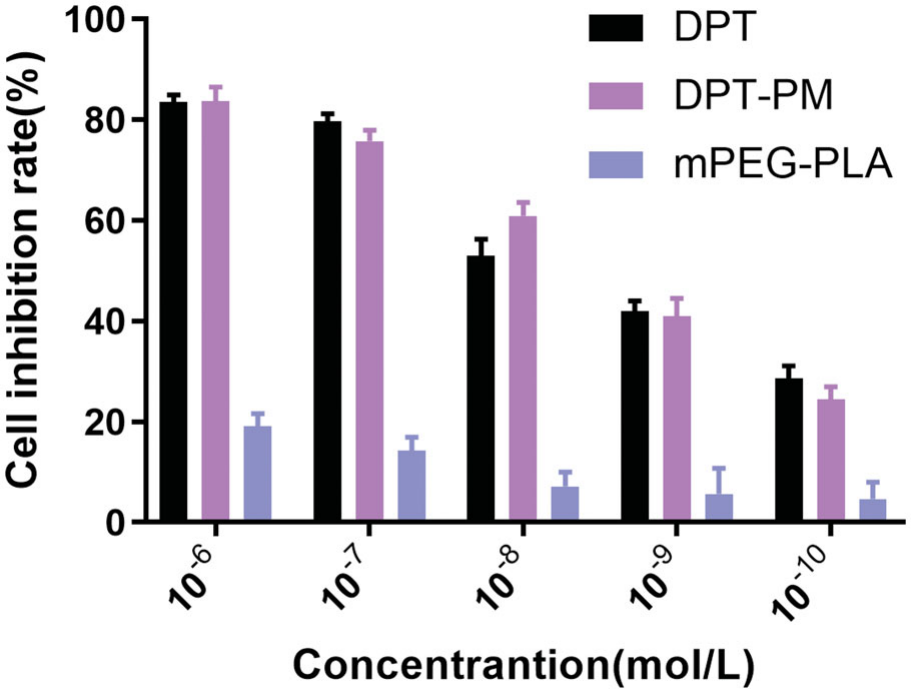
Cytotoxicity of PM, DPT and DPT-PM on Hella cell line (*n* = 3, mean ± SD).

The cytotoxicity level of blank PM, DPT and DPT-PM were shown in Figure 6. Concentration level of all 3 samples was diluted from 10^−6^ to 10^−10  ^mol/L and incubated with Hella cell line for 24 h. The cytotoxicity level of DPT and DPT-PM at 10^−6 ^mol/L concentration was monitored 83.57 and 83.69%, respectively but for PM, only 19.09% toxicity level was calculated which showed that this micelle is safe. However, when the concentration of DPT-PM was 10^−10  ^mol/L, the cell inhibition rate was slightly lower than that of DPT, and then the cytotoxicity increased with the increment of concentration due to that the poor water-solubility of DPT. The micelles increased the solubility of DPT and enhanced the release rate of DPT in tumor acidic microenvironment and increase the cytotoxicity.

### 3.4. The uptake study

**Figure 7.**
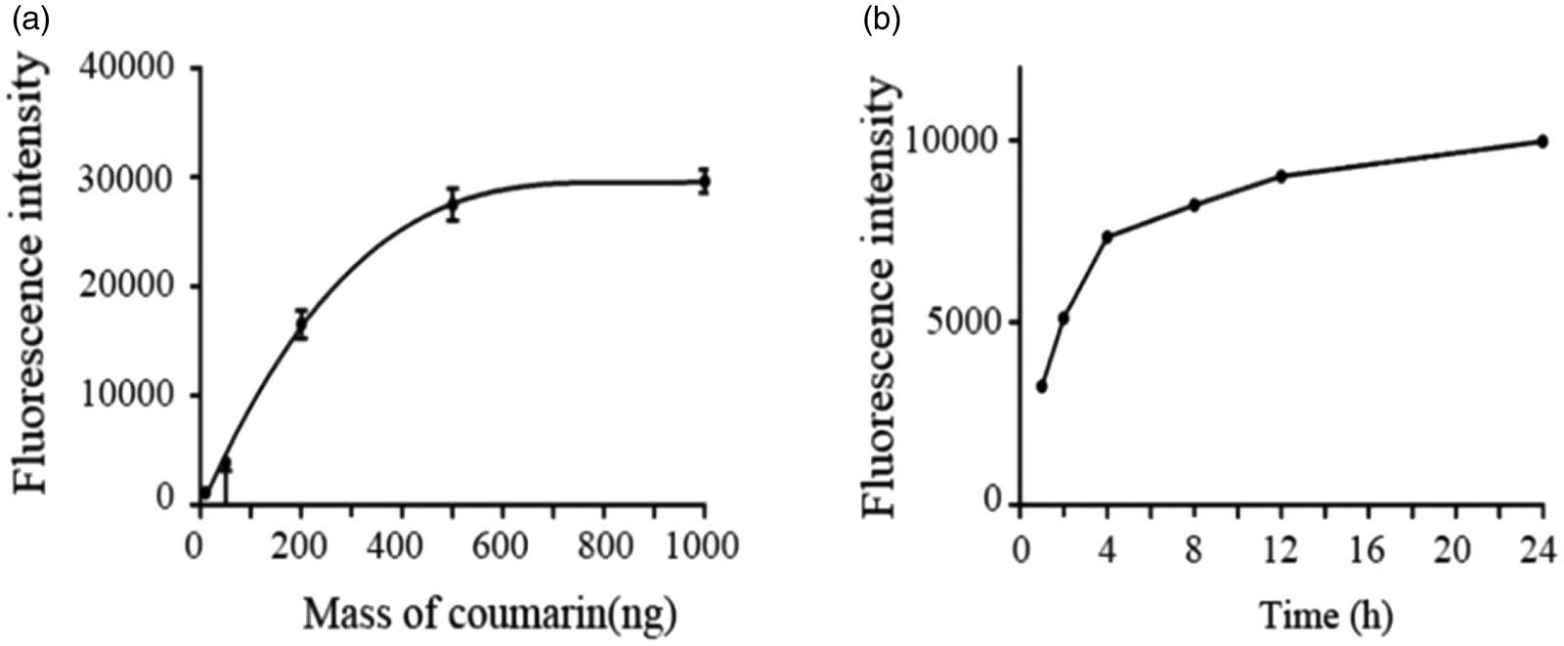
Mean fluorescence intensity of different concentration of mPEG-PLA (a) and at different time (b) (*n* = 3, mean ± SD).

From Figure 7(a), the fluorescence intensity increased with the increment of dose, and the positive correlation trend was obvious which indicates that the uptake of mPEG-PLA micelles was concentration-dependent. But when the dose exceeded more than 500 ng, the cellular uptake was found in saturate level. In addition, Figure 7(b) showed that when the micelles were incubated with cells for 1 h, the fluorescence intensity was only 3200 AU. Then, the results showed that the fluorescence intensity increased rapidly within 4 h, and the fluorescence intensity did not change significantly after 12 h, indicating the uptake for the micelles became saturated after 12 h.

**Figure 8.**
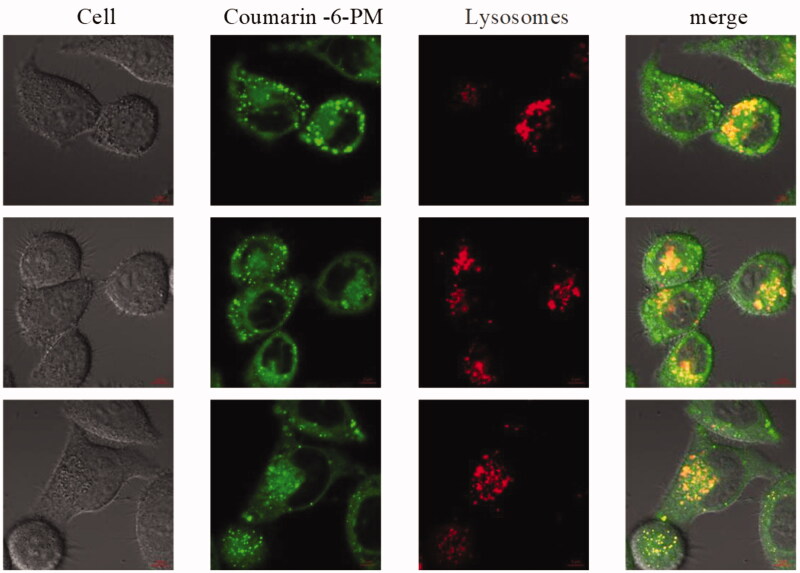
The cell uptake of micelle entrapped coumarin-6 under fluorescence microscope.

Based on the uptake study, the laser confocal scanning was conducted and the scanned images were shown in Figure 8. From the obtained image, it can be seen that PM can stay inside the lysosome for 1–4 h which indicates the uptake of PM inside the cell was through the endocytosis path.

In Summary, the cellualar uptake was investigated by coumarin 6 loaded mPEG-PLA micelles at different dosages and a different time. Compared to DPT, DPT-PM of Hela229 cells had higher ability to kill, while mPEG-PLA showed a higher level of security. The cellular uptake experiment showed that mPEG-PLA micelles can be uptaken by Hela229 cells, and uptake amounts increased with the increase of dosage and time before reaching the saturation. Meanwhile, Hela229 cells in situ nude mice were used as animal models with DiR fluorescent probes to investigate DiR-mPEG-PLA micelles tumor targeting and *in vivo* characteristics of tissue distribution. The result showed that mPEG-PLA micelles had great tumor targeting ability and could accumulate in the tumor. mPEG-PLA micelles also can prolong the time of drug treating time in the tumor through enhanced permeability and retention (EPR) effect.

### 3.5. Tissue distribution

**Figure 9.**
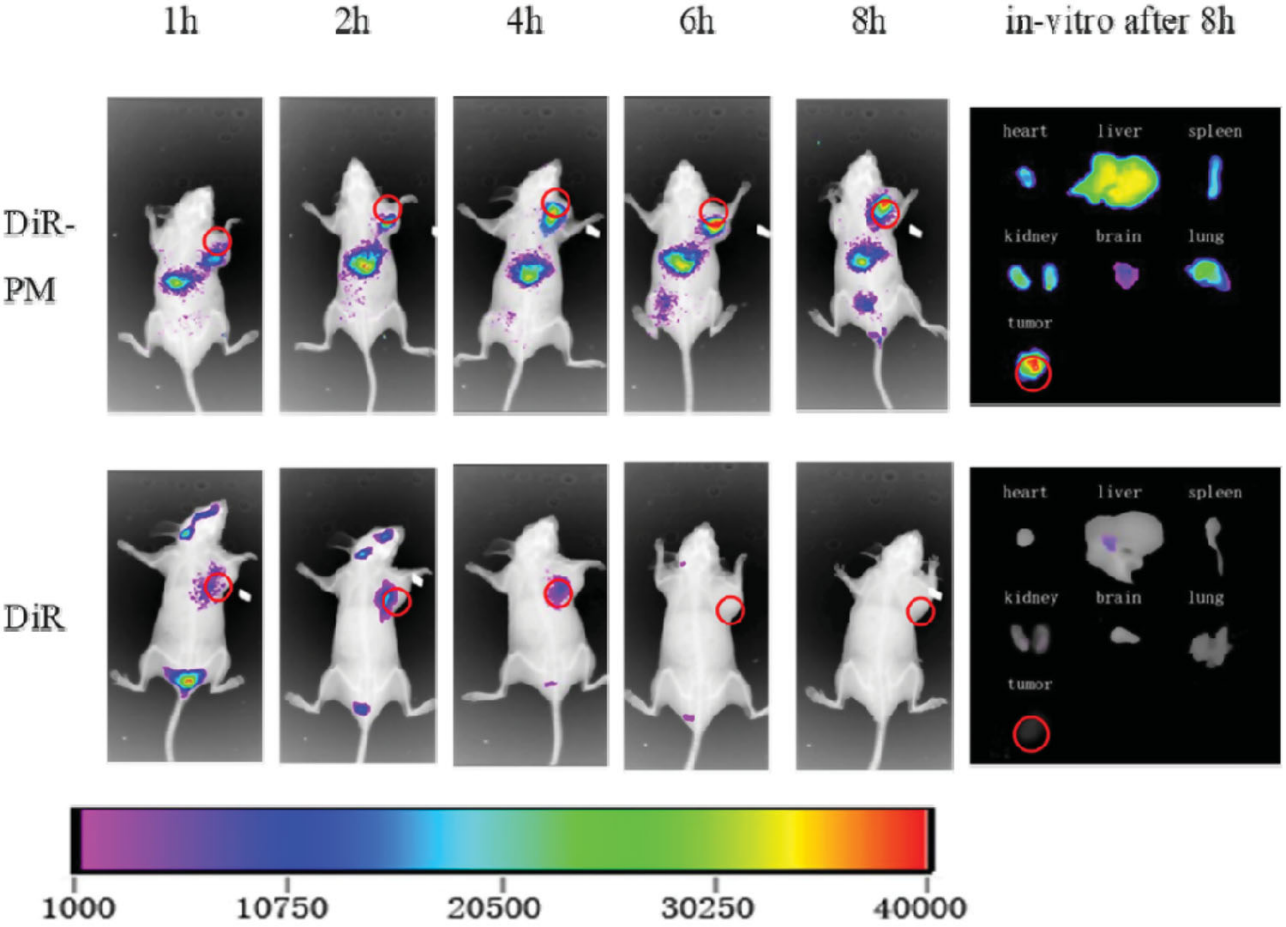
IVIS images obtained after i.v injection of DiR-PM and DiR in tumor bearing mice and *in-vitro* images of major organs after 8 h.

To confirm the mPEG-PLA mediated bio-distribution of DPT, DiR loaded mPEG-PLA was prepared. Then after pre-diluted solution of DiR-mPEG-PLA and DiR in saline were injected into the tumor bearing nude mice via tail vein. After the injection of both preparations, mice were imaged under IVIS at pre-defined time i.e. 1, 2, 4, 6 and 8 h. The obtained IVIS images were shown in Figure 9, the obtained results showed that the fluorescence intensity of DiR in tumor site was gradually increased in the DiR-PM group by the passes of time whereas, in DiR group the fluorescence intensity was only last up to 4 h. Further, to confirm the tissue distribution of DiR, after 8 h mice from both groups were sacrificed and major organs from both groups were harvested and imaged under IVIS. Very strong intensity of DiR was seen in tumor in DiR-PM group as can be seen in Figure 9 (under heading *in-vitro* after 8 h). Whereas in DiR group, no fluorescence intensity was observed. From this bio-distribution study, it was clear that mPEG-PLA has great tumor targeting ability and helps the drug to retain in the tumor for a prolonged period.

### 3.6. Pharmacokinetic study

**Figure 10.**
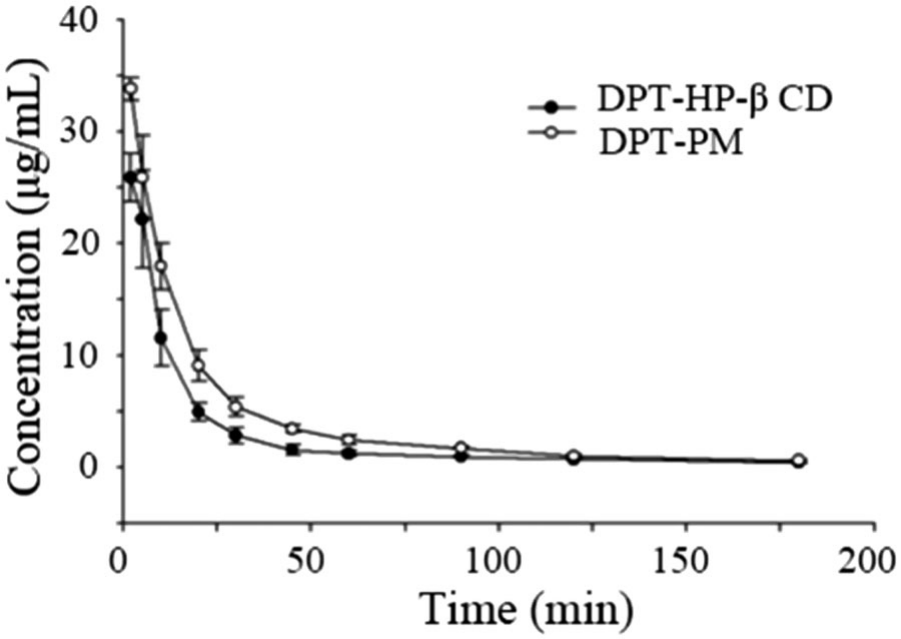
Time courses of DPT plasma level from DPT-MP and DPT-HP-β-CD followed by i.v. administration. Equal dose (15 mg/kg body weight) of DPT was maintained in both formulation (*n* = 6, mean ± SD).

The plasma concentration – time curve of DPT from two formulations followed by i.v.-administration was shown in Figure 10 and the calculated pharmacokinetic parameters were listed in Table 7. From the pK curve, it was clearly seen that the release of DPT from DPX-PM was higher comparing to DPT-HP-β-CD.

**Table 7.** Calculated pharmacokinetic parameters of both formulation followed by i.v administration with same dose (15 mg/kg) of DPT (*n* = 6, mean ± SD).

	*C*_max_	*T*_1/2_	AUC0-t	MRT	CL	Vss
	(μg·mL^−1^)	(min)	(μg·mL^−1^·min^−1^)	(min)	(mL·kg^−1^·min^−1^)	(L·kg^−1^)
DPT-HP-β-CD	25.87 ± 2.13	79.70 ± 3.55	479.65 ± 86.33	56.42 ± 6.23	28.31 ± 5.18	1.60 ± 0.35
DPT-PM	33.83 ± 1.00	93.14 ± 8.18	773.41 ± 97.52	62.29 ± 4.57	18.10 ± 2.57	1.12 ± 0.08

The pK parameters show that the bioavailability of DPT form polymer micelles in plasma was 1.6 times higher than that from cyclodextrin inclusion complexes, indicating that the use of polymer micelles as a carrier can significantly increase the bioavailability of DPT. Moreover, the half-life (*T*_1/2_) of the micelle group was prolonged, plasma clearance was only 63% of the cyclodextrin group and the apparent volume of distribution was 56% of the cyclodextrin group. These results indicated that the mPEG-PLA micelles can prolong the half-life of DPT in plasma and reduce the plasma clearance rate. And can help DPT retain for a prolonged time in the circulatory system for a prolonged therapy.

## 4. Discussion

In this study, the deoxypodophyllotoxin loaded polymeric micelles (DPT-PMs) were successfully prepared by means of solvent evaporation-film dispersion method in the presence of DPT and mPEG-PLA. The TEM and DLS results indicated that the sizes of DPT-PMs were kept below 30 nm with a relatively narrow size distribution. The size of micelles was below the pore size of the permeable vasculature found in many solid tumors, indicating that DPT-PMs could be able to selectively accumulate in solid cancer via the EPR effect (Davis et al., 2008; Fang et al., 2011). This also could explain the mPEG-PLA micelles accumulation in tumors in tissue distribution study.

The DPT-PMs were solidified through lyophilization, which increases the stability of the structure and maintains particle integrity over days. The freeze-dried powder exhibited white pie-solid without collapsing, and the particle size of DPT-PMs reconstituted with water was about 20–35 nm. The entrapment efficiency of reconstituted solution was 98%, which shows no differences with the micelles before lyophilization. Based on the analyses of DSC, it was suggested that DPT was coated by mPEG-PLA in amorphous form.

The release behavior of DPT from the micelles exhibited a biphasic pattern characterized by a fast initial release during the first 10 h, followed by a slower and continuous release of the drug up to 72 h. The burst release of DPT could be the result of the dissolution and diffusion of the drug that was poorly entrapped in the micelles, while the slower and continuous release may due to the diffusion of the drug-loaded in the micelles core. The release behavior of DPT-PMs was pH-dependent, and the sustained release effect of DPT-PMs in acid medium is better than that in neutral medium. Its release mechanism was non-Fick diffusion and fitted with the Peppas–Sahlin equation.

In vitro antitumor activity suggested that mPEG-PLA micelles had satisfactory safety and DPT-PMs could be internalized into the cells efficiently. The cellular uptake of mPEG-PLA micelles was time and dose-dependent. A comparison of in vitro cytotoxicity of free DPT, DPT-PMs enhanced cytotoxicity against Hela229 cells, which would be attributed to the higher internalization of mPEG-PLA micelles than that of free DPT. Meanwhile, Hela229 cells in situ nude mice were used as animal models with DiR fluorescent probes to investigate DiR-mPEG-PLA micelles tumor targeting and in vivo characteristics of tissue distribution. The result showed that mPEG-PLA micelles had great tumor targeting ability and could accumulate in the tumor. mPEG-PLA micelles also can prolong the time of drug treating time in the tumor through enhanced permeability and retention (EPR) effect. Apart from the advantages in delivery, the mPEG-PLA micelles reveal favorable safety for systemic administration. The mPEG-PLA composed of degradable polyethylene glycol and poly D, L-lactide, which were approved by the FDA as an excipient in therapeutic agents (Yang and Kao, 2006). Furthermore, the drugs aggregation and prolonged drugs circulation induced by mPEG-PLA could efficiently reduce the side effects caused by DPT.

It is well known that the surface property of nanoparticles is one of the main factors affecting blood clearance. The micelles with PEG shell can resist nonspecific adsorption of proteins and thus can provide stealth property during blood circulation. Our pharmacokinetic results not only proved the ability of long blood circulation of mPEG-PLA micelles but also displayed the higher bioavailability of DPT-PMs. Hence, all results confirm that the DPT-PMs has better therapeutic efficacy on tumor and lower systemic toxicity than the free DPT.

## 5. Conclusions

In summary, DPT-PM micelle was prepared by solvent evaporation-film dispersion method using mPEG-PLA co-polymer. The used PM has excellent drug loading capacity and selectively release loaded drug in an acidic environment. As in tumor, the cytosolic environment is acidic so this PM can selectively deliver loaded drugs in the tumor without effecting normal cells were pH level is almost neutral. The cellular study also confirmed its selectivity. Further, from *in-vivo* pK study and anti-tumor efficacy study, it was proved that mPEG-PLA co-polymer is a promising carrier. It can selectively deliver loaded drug at the target site to improve the bioavailability of poorly water-soluble drugs and retain in circulation for a prolonged period to discharge the drug in a safe and effective manner.
